# Prevalence of back pain in a group of elite athletes exposed to repetitive overhead activity

**DOI:** 10.1371/journal.pone.0210429

**Published:** 2019-01-24

**Authors:** Daniela Fett, Katharina Trompeter, Petra Platen

**Affiliations:** Department of Sports Medicine and Sports Nutrition, Ruhr-University Bochum, Bochum, Germany; University of Illinois at Urbana-Champaign, UNITED STATES

## Abstract

**Background:**

The prevalence of back pain in athletes has been investigated in several studies, but there are still under- or uninvestigated sports discipline like sports exposed to repetitive overhead activity. Elite athletes spend much time in training and competition and, because of the nature of their disciplines, subject their bodies to a great deal of mechanical strain, which puts a high level of stress on their musculoskeletal systems. From this it is hypothesized that elite athletes who engage in repetitive overhead motions experience a higher strain on their spine and thus possibly a higher prevalence of back pain compared with an active control group.

**Objectives:**

To examine the prevalence of back pain and the exact location of pain in a cohort of elite athletes with repetitive overhead activity and in a control group of physically active sport students. Additionally, to examine different characteristics of pain, and to evaluate the influence of confounders on back pain.

**Methods:**

A standardized and validated online back pain questionnaire was sent by the German Olympic Sports Confederation to German national and international elite athletes, and a control group of physically active but non-elite sports students.

**Results:**

The final sample comprised 181 elite athletes of the sports disciplines badminton, beach volleyball, handball, tennis and volleyball and 166 physically active controls. In elite athletes, lifetime prevalence of back pain was 85%, 12-month prevalence was 75%, 3-month prevalence was 58% and point prevalence was 38%; for the physically active control group, these prevalences were 81%, 70%, 59% and 43%, respectively. There was no significant group difference in prevalence over all time periods. The lower back was the main location of back pain in elite athletes across all disciplines and in controls; additionally a distinct problem of upper back pain was found among volleyball players.

**Conclusion:**

Despite the high mechanical load inherent in the sport disciplines included in this study, the elite athletes who engaged in repetitive overhead activities did not suffer more from back pain than the physically active controls. This suggests that other mechanisms may be influencing back pain prevalences in a positive way in these athletes. Furthermore, these disciplines may practice preventive factors for back pain that outweigh their detrimental factors. Therefore, we posit that extensive prevention work is already being implemented in these sports and that there are additional individual protection factors in play. More research is required to explore these suppositions, and should include investigations into which preventive training programs are being used. Nevertheless, in volleyball particularly, a focus on stabilization/preventive training should be applied to the upper back and neck.

## Introduction

Back pain (BP) is a common complaint, affecting between 54% and 90% of the population, including both younger and older people [1]. BP is not only a medical problem, but also a socioeconomic burden. It may lead to limited functioning in everyday life, impairs quality of work, and is the leading cause of activity limitation and work absence. Pain in the cervical, thoracic, and lumbar spine is also a common complaint among elite athletes [2,3]. Unfortunately, there is a paucity of literature about the frequency and cause of these complaints [4]. While the prevalence of BP in athletes has been investigated in several studies, few, if any, have reported the prevalence of BP in sports with repetitive overhead activities. A systematic review of BP in athletes [2] reported on frequently investigated sports like soccer, gymnastics, and rowing, and on under- or uninvestigated sports like badminton and handball.

Although the exact etiology is unknown, BP or especially low back pain (LBP) is considered a biopsychosocial syndrome that is influenced by a variety of factors. The current literature recognizes three categories of potential risk factors: (1) physical factors, (2) psychosocial factors and (3) individual factors. Accepted physical risk factors are lifting and carrying heavy loads, one-sided loads, flexed positions, rotation and extension, inactive or sedentary lifestyle, and physical activity or extreme sports [5–9]. Although physical activity generally seems to reduce the risk of LBP, too much activity appears to increase the risk, as suggested by the U-shaped exposure-response curve [7].

Elite athletes spend much time in training and competition because of the requirements of their discipline, and subject their bodies to a great deal of mechanical strain and thus their musculoskeletal systems to a high level of stress [3]. The repetitive demands that are placed on the spines of elite athletes in their respective sporting movements is a common feature in elite sport. Trunk rotations of all degrees of freedom have been linked with LBP [10]. As stated in Campbell et al. [10], transverse plane rotations of the trunk during preparation in cricket bowling are considered potentially injurious. In other sports, for example in golf, the axial rotation of the trunk while it is laterally tilted is often the main reason for LBP.

In sports disciplines with repetitive overhead activities such as serving or smashing, the spine is subject to special physical stress. The serve involves high trunk motion speeds and imposes spinal loads up to nearly 3000 N [11]. At the end of the backswing, the axial skeleton is in a position of lumbar extension (arch tension) with an accompanying lateral flexion and rotation of the trunk to the side of the impactor/playing arm side [12]. Additionally, the high demands of these sports that involve high training volumes, full tournament schedules, and repetitive high-loading movement patterns (i.e., exertion of the serve motion, quick starts, stops, and changes of direction, jumps and landings), lead to high musculoskeletal stress, which in turn results in increased injury potential [13]. Injuries and pain are often preceded by overload damage as a result of repetitive microtrauma. The probability of spine injury typically depends on the amount of load and the number of repetitions. After many repetitions, a material weakens; with repetitive loads, such as in overhead sports, the spine can be injured by significantly lower forces. However, the repetitive ballistic trunk movements which are commonly required in overhead sport and which have been associated with high frequency of pars interarticularis stress reaction in other populations [14], underpins the likelihood of a mechanical etiology in this elite sports population [15,16]. From this we hypothesized that elite athletes who perform repetitive, sports-specific overhead movements that are likely to be associated with high loads and intensity (i.e., training volume, repetition of movements and intensity), experience a higher strain on the spine and thus possibly a higher prevalence compared with an active control group.

To the best of our knowledge, there is a lack of information providing detailed description of BP in sport disciplines with repetitive overhead activities with special regard to the prevalences at different time periods (lifetime, 12-month, 3-month and point prevalence) and locations on the spine, typical characteristics of pain (i.e., intensity or disability) and confounders on BP. Therefore, the aim of the present study was to analyze the prevalence of BP as well as the exact location of pain in a cohort of elite athletes with repetitive overhead activity and in a control group of physically active sport students (physically active control group, PACG) using a validated instrument with an internationally accepted definition of BP. Additionally, we examined different characteristics of pain, and evaluated the influence of confounders on BP.

## Materials and methods

### Study design

A survey of elite athletes competing at national or international levels was conducted. A link to an online questionnaire was sent via email in January 2015 by the German Olympic Sports Confederation to the approximately 4,000 elite athletes (squad athletes, best athletes in Germany in their age group) from various sports disciplines in their database. The questionnaire was also sent to a group of 253 physically active but non-elite sports students [3].

The survey was available until March 2015. In this study, we only reported on the sports that involve repetitive overhead activities (i.e., badminton, beach volleyball, handball, tennis and volleyball). To increase the final sample size of elite athletes who engage in repetitive overhead activities, a second recruitment appeal was sent to athletes involved in these selected sports.

All participants were informed about the study in a cover letter, and a consent form describing the purposes and procedures of the study was also distributed to them. The study was approved by the regional committee for research ethics of the medical faculty of Ruhr-University Bochum and by the German Olympic Sports Confederation.

### Definition of participants

Elite athletes were defined as squad athletes who were members of their federal sport association or were participants in the first or second national division in their sport. Squad athletes are the best German athletes in their age group and are broadly divided into A-squad (federal level, national teams), B-squad (potential to join the national team), C- and D-squads (excellent new talents; pool of junior players). Their membership in one of these squads does not automatically mean that they are professionals in the sense of being paid. The extent to which or whether they were being paid to participate in sports was not a requirement for inclusion in this study.

A group of physically active sports students were chosen as a control group. Importantly, we suggest that sports students typically execute moderate, varied forms of movements (i.e., as required by different sports disciplines) and engage in about 10 hours of sport a week, most likely within their study program. As Heneweer et al.’s [7] U-shaped curve relationship between activity level and BP indicates, both large and small amounts of sporting or physical activity appear to predispose a person to BP. It is widely accepted that an active sport participation has positive benefits to the health status. Nevertheless, there is no information about the optimal dose effect relationship [3]. Based on the hypothesis that sport with moderate intensity and low specificity prevents BP, the control group was selected to be hypothetically in the optimal ratio between too little and too much physical activity.

### Back pain questionnaire

The design of the study questionnaire was based on validated, standardized and internationally accepted questionnaires. Details of the questionnaire are described elsewhere [3]. It was divided into three parts. The first part was based on the standardized Nordic Questionnaire [17], which includes several questions about BP, including separate questions about the neck, upper back and lower back. The term ‘back pain’ was used if the pain occurred in at least one part of the back (neck, upper back, lower back). Questions relating to pain focused on lifetime prevalence, 12-month and 3-month prevalences and point prevalence, defined as pain during the last 7 days. Pain was defined as pain, ache or discomfort in an area shown on a diagram of the human body. The second part of the survey consisted of the 7-item questionnaire devised by von Korff et al. [18] for grading the severity of chronic pain in terms of intensity and pain-related disability. The score allows BP patients to be classified into one of five hierarchical categories of pain intensity or disability [18]. Additionally, three questions about sport-related disability were added.

- In the past 3 months, how much has BP interfered with your ability to perform your training session?
- In the past 3 months, how much has BP interfered with your ability to take part in competitions?
- About how many days in the past 3 months have you been kept from your usual competitive sport activity (including training and competition) because of BP?

The third part of the survey was a self-developed questionnaire that focused on sporting activity. It was thoroughly pilot-tested. Reliability of the self-developed questionnaire was tested on a sample of 238 students and competitive athletes. The test-retest reliability (cohen`s kappa) was good to excellent, indicated by kappa values between 0.73 and 0.93. It covered questions about individual sports disciplines, competition level, volume of training and competition, and annual training schedule. More specifically, questions included:

- What kind of sport are you doing?
- How many years have you been practicing your main sport (playing experience)?
- What is your current level of competition?
- How often and how long do you train during the week?
- In which kind of period of your annual training schedule are you currently?
- How often do you compete in your sport per year?

### Statistical analysis

Statistical analysis was undertaken using SPSS software (version 23, IBM, Armonk, US). Respondents’ characteristics were expressed as means and standard deviations. All prevalence data and response rates were rounded to the nearest integer. Group means were compared using unpaired *t*-tests for age, height, weight, training volume, playing experience and number of competitions, and using Pearson’s chi-squared test for sex. Differences in the prevalence of BP between athletes competing in different sports and controls were assessed using the chi-squared test. An unpaired *t*-test was used to determine differences in the intensity and disability of BP between elite athletes and the PACG. Differences in BP severity were tested with the chi-squared test of goodness of fit. Correlations among BP and age, training volume, playing experience and number of competitions were calculated using point-biserial correlation. Binary logistic regression analyses were used to examine the interaction of different confounders for BP in elite athletes, using lifetime and point prevalence as outcomes (no/yes). As independent variables training volume, competition level, playing experience and number of competitions were used as potential confounding variables. Additionally, binary logistic regressions were used to assess whether anthropometrics (age, sex, height and weight) were potential predictive factors for developing BP. Odd ratios with 95% confidence intervals are reported. Statistical significance was defined as *p* < .05.

## Results

A total of 181 elite athletes who engage in repetitive overhead motions and 166 physically active students participated in this study. Survey responses from 1,237 elite athletes and 187 physically active controls were received (response rates of 31% and 74%, respectively). Among elite athletes, only squad athletes (A, B, C, or D grades) and athletes participating in the first or second national divisions of the sports disciplines of badminton, beach volleyball, handball, tennis, or volleyball who were at least 13 years old were included in the analysis. This led to the exclusion of 123 athletes owing to their lower competition level or younger age, and to the exclusion of 1,013 athletes who were not involved in sports with repetitive overhead activities. We also excluded 21 respondents from the PACG who reported being competitive athletes at an elite squad level. At this stage, the sample comprised 101 elite athletes who engage in repetitive overhead activities and 166 PACG respondents. To increase the final sample size of the elite athlete group, a second recruitment appeal was sent to these athletes involved in the selected sports; another 80 athletes answered the questionnaire. So final sample consisted of 181 elite athletes with repetitive overhead activities Sample characteristics are shown in Table 1. Statistically significant between-group differences (PACG vs. athletes) were observed for mean age (p < .001) and training volume (p < .001). The proportion of males in the PACG was also significantly higher (p < .001) than in the group of elite athletes (75% compared with 54%).

**Table 1:** Subjects characteristic.

	Controls *(N = 166)*	All athletes *(N = 181)*	p-value*	Badminton	Beach volleyball	Handball	Tennis	Volleyball
				*(N = 23)*	*(N = 10)*	*(N = 56)*	*(N = 39)*	*(N = 53)*
***Anthropometrics***								
Age [years]	21.2 ± 2.0	19.7 ± 4.7	< 0.001	22.5 ± 4.3^d^^,^^c^	22.3 ± 5.3^d^	19.5 ± 3.8^d,^^e^	15.2 ± 1.8^e^	21.5 ± 4.8
(range)	(18–28)	(13–34)		(16–31)	(17–34)	(14–32)	(13–19)	(15–34)
Height [cm]	180.1 ± 8.9	181.9 ± 12.3	0.121	177.9±10.8^b^^,^^e^	189.2 ± 7.5^c^^,^^d^	179.2 ±9.1^d^^,^^e^	172.8 ± 12.2^e^	191.6 ± 9.2
Weight [kg]	74.0 ± 10.3	74.5 ± 14.8	0.739	71.1 ± 10.9^d^^,^^e^	77.9 ± 9.9^d^	76.0 ± 12.7^d^^,^^e^	60.6 ± 12.7^e^	83.4 ± 13.0
Gender (m/f) [%]	74.7/24.1	54.1/45.9	< 0.001	52.2/47.8	70.0/30.0	46.4/53.6	59.0/41.0	56.6/43.4
***Training and competition data***								
Training volume [h/wk]	10.8 ± 5.0	17.3 ± 6.6	< 0.001	19.8 ± 7.5^c^	19.2 ± 8.0^c^	13.2 ± 4.2^d^^,^^e^	17.2 ± 4.9^e^	20.5 ± 7.0
(range)	(3–40)	(2–42)		(6–32)	(2–30)	(5–28)	(8–26)	(8–42)
Number of competitions [N/year] (range)	-	35.3 ± 16.9	-	23.6 ± 10.6^c^^,^^e^	20.7 ± 7.5^c^^,^^e^	42.8 ± 16.5^d^	30.2 ± 15.7^e^	39.1 ± 16.6
		(1–80)		(8–50)	(14–30)	(1–75)	(12–80)	(3–70)
Playing experience [years] (range)	-	11.6 ± 4.5	-	14.2 ± 5.2^b^^,^^d^^,^^e^	8.1 ± 3.4^c^	13.3 ± 3.9^d^^,^^e^	9.4 ± 2.5	11.1 ± 5.0
		(4–27)		(7–27)	(4–16)	(4–23)	(4–15)	(4–26)
***Competition level [%]***								
A-squad	-	13	-	0	20	9	13	21
B-squad		12		26	20	6	13	9
C-squad		32		22	50	35	32	32
D-squad		13		9	10	11	32	6
1st or 2nd division		30		43	0	40	10	32
***Period of annual training schedule [%]***								
Preparation period	-	21	-	30	80	9	36	8
Spec. comp. prep.		16		17	0	11	26	19
Competition period		55		39	10	70	36	74
Out of competition		9		22	20	13	8	2

* refers to the comparison between controls and all athletes

^b^ significant different to beach volleyball

^c^ significant different to handball

^d^ significant different to tennis

^e^ significant different to volleyball, f = female, m = male, spec. comp. prep = specific competition preparation.

### Prevalence of back pain

Lifetime, 12-month, 3-month, and point prevalence of BP in five different sport disciplines and in the PACG are presented in Table 2. Across all elite athletes, lifetime prevalence of BP was 85%, 12-month prevalence was 75%, 3-month prevalence was 58%, and point prevalence was 38%. In the PACG, these measures were 81%, 70%, 59%, and 43%, respectively. There was no significant group difference in prevalence (over all time periods).

**Table 2:** Prevalence of back pain in different locations at the spine.

	Controls	All athletes	p-value*	Badminton	Beach volleyball	Handball	Tennis	Volleyball
	*(N = 166)*	*(N = 181)*		*(N = 23)*	*(N = 10)*	*(N = 56)*	*(N = 39)*	*(N = 53)*
***Lifetime prevalence* [%]**								
Back	81	85	0.349	87	90	80	77	93^+^^,^^d^
Neck	50	51	0.839	52	60	54	51	45
Upper back	39	34	0.394	22^e^	40	30	28	47
Lower back	71	74	0.428	83	70	75	54^a^^,^^c^^,^^e^	83
*Pain in multiple areas*	*49*	*52*	*0*.*637*	*48*	*60*	*63*^*d*^	*41*	*49*
***12-month prevalence* [%]**								
Back	70	75	0.272	70	80	80	72	74
Neck	39	41	0.606	26^c^	60	48	44	34
Upper back	27	27	0.979	22	30	25	21	34
Lower back	58	61	0.603	70	60	63	51	64
*Pain in multiple areas*	*37*	*38*	*0*.*966*	*30*	*50*	*46*	*36*	*30*
***3-month prevalence* [%]**								
Back	59	58	0.766	48	70	59	56	59
Neck	30	28	0.938	17	40	29	31	28
Upper back	22	18	0.278	9	20	18	13	25
Lower back	46	44	0.799	44	50	48	36	45
*Pain in multiple areas*	*27*	*24*	*0*.*555*	*17*	*30*	*27*	*21*	*25*
***Point prevalence* [%]**								
Back	43	38	0.323	39	60	39	33	34
Neck	22	16	0.200	17	30	14	15	15
Upper back	15	11	0.222	4	10	13	8	13
Lower back	29	27	0.771	30	40	25	23	26
*Pain in multiple areas*	*16*	*13*	*0*.*346*	*13*	*20*	*9*	*13*	*15*

* refers to the comparison between controls and all athletes

^**+**^ significant different to PACG

^a^ significant different to badminton

^c^ significant different to handball

^d^ significant different to tennis

^e^ significant different to volleyball.

Concerning the lifetime BP prevalence for different sport disciplines, volleyball had the highest, followed in order by beach volleyball, badminton, handball, and tennis. Only volleyball showed a significantly higher BP prevalence (p = .045) compared with the PACG. Among the sports disciplines, volleyball’s prevalence was only significantly higher than that of tennis (p = .035).

The highest 12-month BP prevalence was found for handball, followed in order by beach volleyball, volleyball, tennis, and badminton. No differences were found between any of the sports and the PACG group, nor among the sports discipline themselves.

The highest 3-month BP prevalence was found for beach volleyball, followed in order by handball, volleyball, tennis, and badminton. Beach volleyball also had the highest point prevalence, followed by handball, badminton, volleyball, and tennis. No differences were found within the sports or between elite athletes and the PACG.

### Location of pain

Table 2 also presents an overview of the reported location of pain. The distribution of BP location was nearly identical for all elite athletes, for the PACG, and in the different sport disciplines. The low back was the most commonly affected area for all time periods, followed by the neck and upper back. Only for volleyball did the distribution differ: these players showed the same lifetime and 12-month prevalence for upper back and neck pain.

The values of pain prevalence in the different spine locations were very similar among all groups, except in isolated cases. For lifetime prevalence of LBP, significant differences were found between tennis and volleyball (54% vs. 83%, p = .001), between tennis and badminton (54% vs. 83%, p = .029) and between tennis and handball (54% vs. 75%, p = .032). For lifetime prevalence of upper BP, a significant difference was found between badminton and volleyball (22% vs. 47%, p = .027). For 12-month prevalence of neck pain, a significant difference emerged between badminton and handball (26% vs. 48%, p = .045).

### Severity of back pain

**Fig 1:**
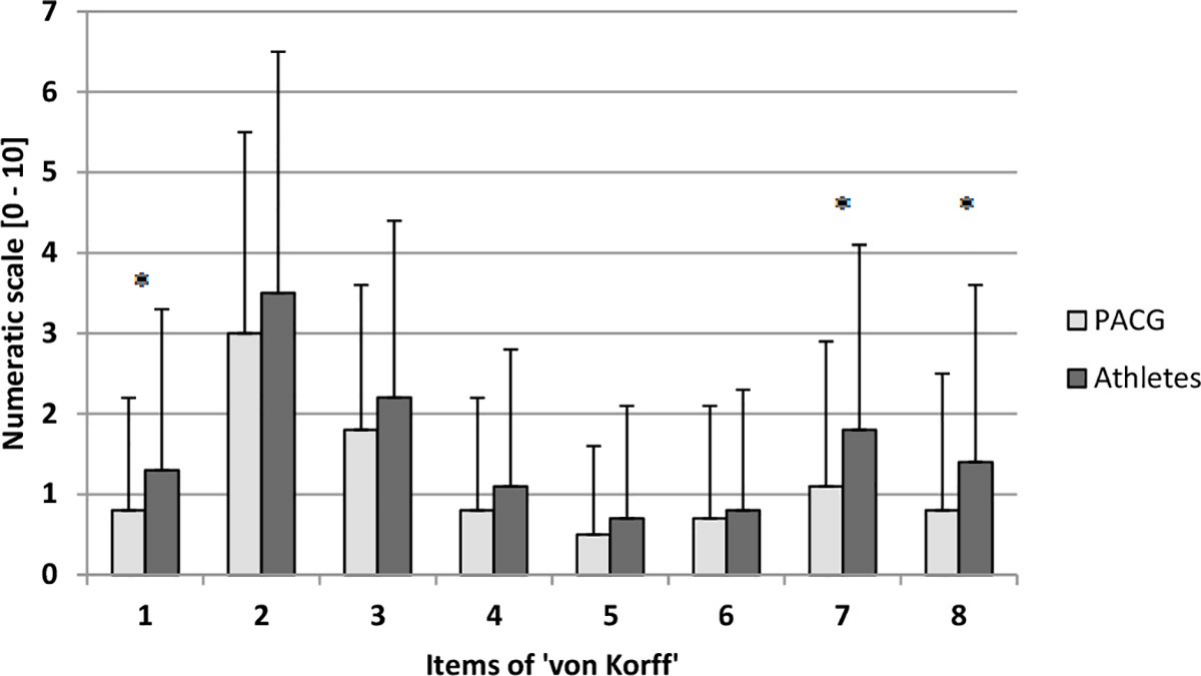
Pain intensity and disability. PACG = physically active control group, 1 = back pain intensity at the present time, 2 = worst back pain intensity during the past 3 month, 3 = average back pain intensity during the past 3 month, 4 = interference of back pain in usual activities (work, school or housework), 5 = interference of back pain with ability to take part in recreational, social and family activities, 6 = interference of back pain with ability to work, 7 = interference of back pain with ability to take part in training, 8 = interference of back pain with ability to take part in competition; * = p<0.05.

Results for BP intensity and extent of BP-related disability of elite athletes and of the PACG are shown in Fig 1. The group of elite athletes showed significantly higher values for the items “back pain intensity at the present time” (p = .014), “interference of back pain with ability to take part in training” (p = .001), and “interference of back pain with ability to take part in competition” (p = .006).

**Fig 2:**
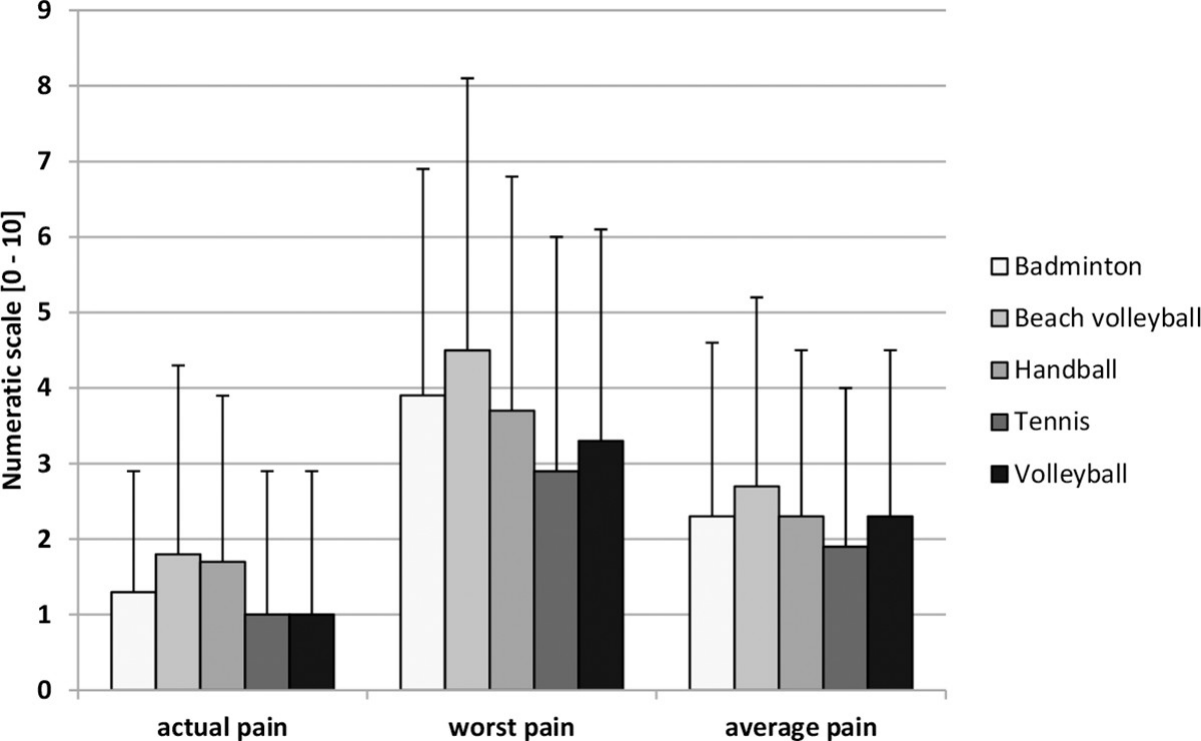
Back pain intensity between different sports disciplines.

BP intensity by sport is shown in Fig 2. Beach volleyball players clearly had the highest values over all items. Results for pain severity are shown in Table 3. Most of the respondents reported grade 0 (no pain) or 1 (low disability—low pain intensity). The distribution of the severity grades between athletes and the PACG differed significantly for grade 2 (elite athletes 12% vs. controls 4%, p = .001). Significant differences were also found between tennis and badminton (grade 0, p = .007), between tennis and volleyball (grade 0, p = .012; grade 1, p = .020), and between badminton and volleyball (grade 3, p = .032).

**Table 3:** Severity grade.

Severity grade	Athletes	Controls	p-value*	Badminton	Beach volleyball	Handball	Tennis	Volleyball
	[%]	[%]		[%]	[%]	[%]	[%]	[%]
**0**	27.1	24.7	0.297	21.7^d^	30.0	26.8	41.0	18.9^d^
**1**	58.0	68.7	0.080	60.9	50.0	57.1	46.2	67.9^d^
**2**	12.2	4.3	0.001	8.7	20.0	12.5	10.3	13.2
**3**	2.2	1.2	0.092	8.7^e^	0.0	3.6	0.0	0.0
**4**	0.0	0.6	0.051	0.0	0.0	0.0	0.0	0.0

* refer to the comparison between controls and all athletes

^d^ significant different to tennis

^e^ significant different to volleyball, 0 = no pain, 1 **=** low disability—low pain intensity, 2 = low disability—high pain intensity, 3 = high disability–moderately limiting, 4 = high disability—severely limiting.

### Back pain and confounders (training volume, playing experience, number of competitions, age, sex)

Training volume was 17.3 ± 6.6 hours per week for elite athletes and 10.8 ± 5.0 hours per week for the PACG. Among the sports disciplines, volleyball had the highest training volume, followed by badminton, beach volleyball, tennis, and handball (Table 1). Across all groups and pain distributions, several correlations between BP and *training volume* were found. In the group of all elite athletes, a positive correlation was observed between lifetime (p = .001, r = 0.255) and 12-month (p = .043, r = 0.154) prevalence of upper BP, and point prevalence of LBP (p = .035, r = 0.160). Several sport-specific correlations emerged between training volume and BP. Badminton and beach volleyball were positively correlated with upper BP. For badminton, the lifetime prevalence of upper BP (p = .041, r = 0.429) was correlated positively with training volume, and for beach volleyball, both lifetime prevalence and 3-month prevalence were correlated positively with training volume (respectively, p = .022, r = 0.707 and p = .043, r = 0.648). For handball, significant positive correlations for the lower back were found for lifetime, 12-month, and 3-month prevalence and training volume (respectively, p = .05, r = 0.266; p = .004, r = 0.379, and p = .007, r = 0.364). For tennis, positive correlations emerged with lifetime prevalence of BP (p = .018, r = 0.381) and with 3-month prevalence of LBP (p = .029, r = 0.354). For volleyball, there were significant positive correlations with lifetime prevalence of upper BP (p = .011, r = 0.360) and with 12-month prevalence of neck pain (p = .048, r = 0.290).

Concerning BP and *playing experience* in elite athletes, in most cases no positive correlations were found. Only in badminton higher playing experiences (in years) are associated with higher levels of upper back and neck pain. There were significant positive correlations between lifetime, 12-month and 3-month prevalence of upper BP and playing experience (p = .035, r = 0.441; p = .007, r = 0.545; p = .019, r = 0.484) and between 3-month prevalence of neck pain and playing experience (p = .032, r = 0.448).

A similar result showed the relationship between BP and *number of competitions*, only in the group of volleyball, 3-month (p = .012, r = -0.360) and point prevalence (p = .004, r = -0.404) of BP showed significant correlations with number of competitions.

In the majority of sports and the PACG, correlations between BP and *age* were absent over all time periods and all spinal locations. Exceptions included a correlation among elite athletes with 3-month prevalence of upper BP and age (p = .013, r = 0.187), and among the PACG with lifetime prevalence of LBP and age (p = .045, r = -0.157). For badminton, there was a correlation between 12-month prevalence of upper BP and age (p = .02, r = 0.482); for tennis, between lifetime prevalence of BP and age (p = .025, r = 0.359); and for volleyball, between lifetime prevalence of neck pain and age (p = .036, r = 0.291).

In the majority of all time periods and pain locations, significant *sex differences* were found in the PACG. In contrast, such a difference was found in elite athletes only for the 12-month prevalence of neck pain (p = .049). Within the sports disciplines, only beach volleyball showed sex differences in the 3-month prevalence of neck pain (p = .011) and upper BP (p = .016).

Logistic regression analysis was performed to assess the effect of several potential risk factors for BP. The analysis should check whether the value of a dependent variable can be predicted by the independent variable. The regression model included training volume, playing experience, competition level, and number of competitions. The omnibus tests of model coefficients investigated, whether the model makes an explanation contribution compared to the prediction. For the outcome ‘lifetime prevalence of BP`the model was significant (χ^2^(4) = 10.08, p = .039). In the multivariate model, training volume and competition level were significant predictors of symptoms. The Odd ratios with 95% CI are presented in Table 4. The correct classification rate was 83%. In total, 83% of individuals were classified by the model according to their actual response. However, it must be considered that the correct classification to the group "lifetime prevalence = no" was very low (1%). In contrast, the correct classification to the group "lifetime prevalence = yes" was very high (99%). The omnibus tests of model coefficients were not significant for the outcome ‘point prevalence`. Therefore, the analysis was not continued. The logistic regression model with the independent variables age, sex, height, and weight could also not contribute to the explanation (p > .05).

**Table 4:** Binary logistic regression analysis for lifetime prevalence of BP.

Variables	p-value	OR	95% CI	
			lower	upper
Training volume	0.011	1.119	1.027	1.219
Competition level	0.023	1.253	1.031	1.521
Playing experience	0.958	0.997	0.903	1.102
Number of competitions	0.291	0.987	0.963	1.011

CI = confidence interval, lower = lower limit, OR = odds ratio, upper = upper limit.

In majority, the results of the bivariate statistics showed only few significant correlations or differences. In the binary logistic regression model, influence of training volume and competition level could be indicated.

## Discussion

The aims of this investigation were to evaluate the prevalence of BP, to examine the exact location of pain, to examine disability (i.e., impact of BP on daily life and sports participation), and to examine the influence of confounders on BP in elite athletes with repetitive overhead activities and in a control group of physically active sport students.

Our main findings were: (a) no significant differences in BP prevalence for the majority of sports; (b) statistically significant lower prevalence and severity of BP for tennis compared with other sports; (c) the lower back as the main location of BP in elite athletes across all disciplines and in the PACG; (d) a distinct problem of upper BP among volleyball players; (e) the highest pain intensity and disability values among beach volleyball players; (f) for elite athletes, little interference of pain with ability to take part in everyday life (work, school, social, or family activity) but greater interference with ability to take part in training or competition; and, (g) an influence of training volume and competition level whereas no influence of age, sex, height, and weight in the group of elite athletes.

### Prevalence of back pain in different sports disciplines with overhead activity

Only a few studies have reported BP prevalences in sports that involve repetitive overhead activities [2]. For badminton, Noormohammadpour et al. [19] found lifetime, 12-month and point prevalences of 62%, 42%, and 13%, respectively. In line with these results, Schulz et al. [20] reported a 1-year incidence of 57%. For handball, Tunas et al. [21] reported lifetime, 12-month and point prevalences of 63%, 59%, and 26%, respectively; in contrast, Schulz et al. [20] documented a 1-year incidence of only 29%. For tennis, lifetime prevalences between 50% and 53% have been reported [19,22]. Noormohammadpour et al. [19], moreover, reported 12-month and point prevalences of 31% and 19%, respectively. For volleyball, lifetime prevalences of 63% and point prevalences between 20% and 34% have been found [19,23,24]; however Schulz et al. [20] reported a 1-year incidence of 79%. To the best of our knowledge, no data have been recorded for beach volleyball. Together, these data show different and controversial results concerning prevalence rates, especially for handball and volleyball.

On the one hand, prevalences in our investigation seem to be higher than those in the aforementioned literature concerning BP prevalences of overhead sports disciplines despite using the same questionnaire for part of the data gathering. Tunas et al. [21], Swärd et al. [22], and Bahr [23] also used the Nordic questionnaire for investigating BP prevalences in overhead sports disciplines and reported lower BP prevalences compared to our results, although the samples had similar age range and performance level. The reasons for the different prevalences can be manifold, as there are a variety of factors (individual, physical and psychosocial factors) influencing the development of back pain that are not apparent from the comparison of the studies.

On the other hand, prevalences in our investigation seem to be much lower than those of other sports disciplines. In a study of BP prevalences of 1,114 German elite athletes [3] much higher BP prevalences were found over all sports disciplines compared to our results. Mean lifetime, 12-month, 3-month, and point prevalences were 89%, 81%, 68%, and 49%, respectively. This study also reported large differences among the sports disciplines, with lifetime prevalences from 56% for triathlon to 100% for fencing, diving, and water polo. Twelve-month prevalence ranged from 44% for triathlon to 96% for fencing; 3-month prevalence from 38% also in triathlon to 90% in taekwondo; and point prevalence from 28% in volleyball to 74% in water polo [3].

The prevalences in our investigation of sports disciplines with repetitive overhead activities are quite similar. On a purely descriptive basis, beach volleyball and handball showed the highest prevalences and pain intensity rates, and tennis the lowest rates. Statistically significant differences between the sports disciplines were only found in isolated instances. Notably, tennis players had significantly lower prevalence of LBP than did volleyball, badminton, or handball players. However, it should be noted that tennis players were also youngest (mean age 15 years) and lightest. Being younger and having a lower body mass are both associated with lower BP prevalence rates [25]. It is therefore difficult to determine whether this result was an effect of sport, or of other confounders.

### Prevalence of back pain between all athletes and controls

The present study shows no differences in BP prevalence between elite athletes of sports with repetitive overhead activities and a PACG. Some researchers have hypothesized that elite athletes would have a higher prevalence of BP, because of the degree of stress on the musculoskeletal system during highly competitive levels of sport [3,19] particularly when compared with participants such as the PACG, which have activity levels closer to optimal according to the U-shaped curve [7] mentioned earlier. Despite the elite athletes’ subjection to higher mechanical loads from their high frequency of jumps and landings, extreme posture positions (i.e., repetitive lumbar flexion, hyperextension, and rotation), and other high-loading movement patterns (i.e., exertion of the serve motion, quick starts, stops, and changes of direction), which are associated with higher incidences of BP [26], in our investigation elite athletes had no higher BP prevalences compared with an active control group. It may be that other mechanisms can influence prevalences in a positive way. For example, some important aspects of elite sport are prevention programs, core stability, and recovery. At this point, we can only assume that a lot of prevention work is already being implemented in these sports and that there may also be other individual protection factors in play. This could help explain why the BP prevalences amongst elite athletes are not higher than those in the PACG, despite their presumably higher mechanical loads. Thus, it is necessary to clarify in further research which preventive training programs are being executed in these elite sport disciplines. Future studies, moreover, should focus even more on each athlete to determine their individual factors (i.e., individual stress sensation, recovery-stress relation, and use of stabilization exercises). To understand the extent to which training content influences BP, this analysis should be conducted at the club or coach level to determine differences in BP between members of same club or players of the same coach. Another factor that needs to be discussed with regard to the results is the choice of the control group. It might be that the PACG is not in the optimal ratio between too little physical activity and too much physical activity concerning BP prevention. Comparing the prevalences of the PACG with data from the general population, they are still quite high [1].

### Location of pain

Another interesting finding is the higher prevalence of upper BP in volleyball. Over all time periods, volleyball showed the highest upper BP values compared with the other sports disciplines. Volleyball also produced a different distribution of affected back areas. In the other four sports disciplines the low back was the most commonly affected area, followed by the neck and upper back, while volleyball players showed the same prevalences for the upper back as for the neck. This may be because of the flexed position of the spine and simultaneous hyperextension of the neck when players receive the ball and in defending situations. Dalichau and Scheele [12] evaluated the influence of the motoric demands of competitive volleyball on the thoracolumbar spinal configuration. They reported that, compared with a control group, the angle of thoracic kyphosis of the athletes was significantly increased in the sagittal projection. They suggested that particularly the sports-specific skills such as hitting and serving seem to influence the spinal configuration in a special way. In another study, Bartolozzi et al. [27] investigated the prevalence of degenerative changes of the intervertebral discs, and showed a prevalence among competitive volleyball players of 44%, whereas the rate in the control group was only 20%. It was striking that players who were repeatedly subjected to high mechanical stresses in the spinal column as a result of overstressing during their training showed significantly more frequent structural changes to the discs than did players who followed a continuous training with regular stretching exercises, extensive warm-up and regeneration contents integrated into their training process [27]. Radiographic evidence of disc degeneration is more prevalent in athletes than in non-athletes; however, it remains unclear whether this correlates with a higher rate of BP [28], or whether it threatens the athlete’s career [29]. The results suggest that the *intensity* of the mechanical loadings applied in the training and the presence of suitable stabilizing and regenerative measures determine spinal configuration, the degree of degenerative spinal changes and pain characteristics. These aspects should be taken into account in the preventive training of athletes.

### Severity of back pain

BP is one of the most common reasons for missed playing time in athletes [28]; it can be severely detrimental in athletes’ efforts to participate in their daily training or competition. In the present study, athletes felt significantly more impaired in terms of training and competition compared with controls. However, it must be noted that training and competition are not important for the control group, because its members do not participate in competitive sports. Additionally, even minor pain can hamper an athlete’s performance; therefore, pain has a greater impact at the elite level [29]. However, while athletes feel impaired in their sport performance by the pain, they are not inhibited during everyday activities. Concerning pain severity grades, our data indicate a significantly higher percentage of grade 2 among the athletes compared with the PACG, which means they have higher levels of pain but still feel unimpaired in everyday life.

With respect to differences in severity grades between the sport disciplines, tennis players showed higher rates of grade 0 (no pain) compared to other sports disciplines, which is in line with the lower prevalence rates. As discussed above, this outcome may reflect the lower age, lighter weight, other training intensities and contents, and/or recovery-stress ratio.

### Back pain and confounders

Another point frequently discussed in the context of BP is athletes’ weekly *training volumes*. Both high and low amounts of sporting activity appear to predispose individuals to BP, according to the U-shaped exposure-response curve [7]. Elite athletes spend a considerable amount of time in training and competition. In the literature training volume is a controversial discussed risk factor for athletes. In studies of Tunas et al., Bahr et al., and Maselli et al. [21,30,31] no relationship between BP and training volume was found, whereas in other studies this relationship could be confirmed [32,33]. In our investigation, due to the logistic regression, the relationship between training volume and BP could be confirmed and also in the bivariate statistic, several positive correlations were found. In the present investigation, the average training volume of elite athletes was 17 hours per week, with a range from 13 (for handball) to 21 (for volleyball) hours per week. Compared with endurance sports, game sports have lower training volumes; nonetheless, our results show that for badminton, volleyball, and beach volleyball a higher training volume seems to negatively influence upper BP. Possibly this can be attributed to a higher mechanical load on the upper spine due to higher training volumes. On a purely descriptive basis badminton, volleyball, and beach volleyball had higher training volumes compared to handball and tennis. Significant differences are shown in Table 1.

Nevertheless, Fett et al. [3] reported large differences in prevalence of BP between different disciplines with similar training volumes; they asserted that the intensity and content of training, and the physical and psychological constitution of an athlete, are likely additional influences. These factors may also impact long-term pain outcomes [19]. In relation to our results, although training volume came out to be a significant predictor of BP in binary logistic regression and elite athletes had a significantly greater training volume than controls, the prevalence of BP did not significantly differ between elite athletes and controls. This additionally underlines that the development of BP is a biopsychosocial process of multiple factors and not due to a single factor.

In addition to the training volume the *playing experience* must also be considered, because the high loads have to be endured for years. This aspect has been examined in several studies with controversial results. In studies of Cali et al. [34], Maselli et al. [31] and van Hilst et al. [33], no relationship between playing experience and BP could be found. Otherwise Erikson et al. [35] found an effect, as well as the results of this study in terms of upper BP and playing experience.

Furthermore, *age* is a frequently discussed confounder for BP. In the general population, there is lower prevalence of BP in children compared to adults. BP rises with age and peaks at 55–64 years [36]. In addition, earlier studies have shown that some demographic factors such as age can increase the probability of LBP in athletes [37]. In our investigation, such a relationship wasn’t found; however, this might be explained by the relatively small age range of our respondents.

Regarding *sex*, in the general population, BP is reported more commonly in females than in males, and different physiological, social and educational explanations have been discussed [38–41]. However, in athletes the relationship between BP and sex is controversial. Some studies have reported that adolescent and adult female athletes are more likely to report BP [38,39,42,43], while others have found the opposite pattern [22,23,44]. Sex differences in the prevalence of musculoskeletal pain in elite athletes might be influenced by different factors. In some disciplines, male athletes might tolerate greater loads because of their higher training volume or because of inherently higher loads during strength training, or because of differences in basic rules [3]. For our group of elite athletes, we couldn't found this influence.

### Limitations

There are limitations that should be considered regarding the present results. Our findings may have been influenced by recall bias, which is a particular concern in any retrospective cross-sectional study. There may also have been a response bias caused by acquiescence, socially desirable responding or extreme responding [3]. Athletes and controls with BP may have been more likely respond to our survey, so our findings should be interpreted with caution.

Any comparison of BP between elite athletes and the general population, or even a PACG, is difficult. Experience of pain may be influenced by factors like susceptibility, motivation and physical activity. On the one hand, minor pain may be provoked by vigorous body movements that hamper athletic performance, thereby rendering pain more influential for athletes than for the general population. On the other hand, a well-motivated athlete may ignore even severe pain to maintain or improve athletic performance [29].

Also, the analysis of prevalences in different sports disciplines should be interpreted carefully, because it may have been affected by sample size. The comparison between elite athletes and the PACG must be interpreted in the context of the significant between-group differences in age, sex and training volume. Further it is speculative, if this sample is representative of all German sportspersons in the examined sports.

Additionally, we added three questions about sport-related disability to the questionnaire by von Korff et al. [18] (for grading the severity of chronic pain in terms of intensity and pain-related disability), which may have hampered the psychometric properties of the instrument.

## Conclusion

BP is a common complaint in the general population as well as in German elite athletes. However, while the mechanical load is very high in the sports disciplines included in this study, elite athletes reported no more BP compared to the PACG. We posit that other mechanisms may influence prevalences in a positive way. For example, some important aspects of elite sport are prevention programs, core stability, and recovery. At this point, we can only assume that a lot of prevention work is already being implemented in these sports, thus, it is necessary to clarify in further research which preventive training programs are being executed in these elite sport disciplines, and that there may also be other individual protection factors in play. This could help explain why the BP prevalences amongst elite athletes are not higher than those in the PACG, despite their presumably higher mechanical loads. Further research is required to understand these unexpected findings. Nevertheless, our findings suggest that prevention and regeneration strategies, as well as an emphasis on regulating exercise intensity, should be prioritized in considerations of everyday training. In volleyball particularly, a focus on stabilization/preventive training should be applied to the upper back and neck.

The results of the current study could provide the first steps toward developing sport-specific clinical guidelines for addressing BP in elite athletes. In future studies in these sports disciplines, researchers should investigate mechanical load on the one hand and preventive measures that could avert the development of BP on the other. In addition to physical risk factors, individual and psychosocial factors should also be considered. This would offer the opportunity to prevent BP in elite athletes and enhance their overall health.

## Supporting information

S1 File Data availability. (SAV)
